# Women Deprivation Index and Family Planning Utilisation in Urban Geography of West African Countries

**DOI:** 10.3389/fgwh.2021.656062

**Published:** 2021-06-16

**Authors:** Akanni Ibukun Akinyemi, Jacob Wale Mobolaji, John Olugbenga Abe, Elhakim Ibrahim, Olutoyin Ikuteyijo

**Affiliations:** ^1^Department of Demography and Social Statistics, Obafemi Awolowo University, Ile-Ife, Nigeria; ^2^Department of Demography, The University of Texas at San Antonio, San Antonio, TX, United States; ^3^Swiss Tropical and Public Health Institute, Department of Epidemiology and Public Health, University of Basel, Basel, Switzerland

**Keywords:** family planning, deprivation, urban, contraception, long-acting reversible contraceptives, Africa

## Abstract

Inequalities in health care utilisation and outcomes vary significantly across geographies. Though available evidence suggests disparity in contraceptive uptake in favour of urban compared with rural geographies, there are unassessed nuances among women in urban communities. This study examines some of these disparities within the context of socioeconomic deprivations and family planning utilisation among urban women in West Africa. A secondary analysis of the most recent Demographic and Health Survey dataset of five selected West African countries was conducted, using pooled data of 21,641 women aged 15–49 years. Associations between family planning utilisation and women's deprivation status were investigated using a binary logistic regression model. The findings show that more than one-quarter of the women were severely deprived across the countries except Senegal (17.4%), and the severely deprived consistently have relatively low contraceptive prevalence rates (CPR) (16.0–24.3%) compared with women with no/low deprivation across the countries except Senegal (39.8%). The results for long-acting reversible contraceptives (LARC) were not consistent across the five countries: whereas, LARC utilisation was lower among severely deprived women in Nigeria (9.1%), Guinea (9.6%), and Mali (19.3%), utilisation was similar across the deprivation groups in Benin and Senegal. In the multivariable analyses, the log-odds of modern contraceptive utilisation decreases by 0.27 among the moderately deprived (ß = −0.27, SE = 0.05, *p* < 0.01) and by 0.75 among the severely deprived women (ß = −0.75, SE = 0.05, *p* < 0.01) compared with those with no/low deprivation, with variations across the countries. Similarly, the log-odds of LARC utilisation decreases by 0.44–0.72 among the severely deprived women compared with those with no/low deprivation across the countries except Senegal. This study concluded that family planning intervention programmes and policies need to underscore the deprivation context of urban geographies, particularly among women living in informal settlements.

## Introduction

Sub-Saharan Africa (SSA) is regarded as the world's most rapidly urbanising region (1). With the growing urban population, there are concerns about the quality of life, and sexual and reproductive health outcomes in the region. Family planning has been identified as a major intervention in improving maternal and child health outcomes as well as that of the family (2–5). However, available evidence across the region suggests that contraceptive uptake is still very low (6–9) and disproportionately in favour of urban geographies (2, 10–12).

Studies relating women empowerment and socioeconomic status to family planning utilisation have focused more on women's economic and educational status (13, 14). However, the urban living condition and configuration suggest multiple levels of deprivation beyond the narrow concepts of economic and educational status. There are very high variations in contraceptive uptake within the urban geographies that are related to space and communities (3, 4, 15–18). Though individual factors play important roles in poor uptake of contraceptives, intended users may lack access to the desired method due to lack of information and intense poverty associated with their geographical location. Studies have shown wide differentials in family planning utilisation among urban dwellers (19) and by women's empowerment levels (9). The analysis of the United States Agency for International Development (USAID) on 47 developing countries attested to the wide inequalities in service use due to women's inability to access and afford services, low status of the females, and cultural norms (20). Despite several initiatives and investment to improve family planning uptake, these inequalities have significant influence on service utilisation.

However, the urban setting presents different typologies and contexts, which are erroneously assumed as homogenous in most analysis (21). The context for women living in urban areas in West African countries differs and varies across the extent of deprivation. For instance, women living in informal urban settings are usually associated with extreme poverty, low level of education, poor health care services (15, 22), among others. Obviously, health care utilisation of residents in the informal setting is incomparable with that of their counterparts in formal urban areas.

The underpinning analytical framework is guided by the need to expand the social analysis of health behaviour beyond the limited individual factors (23). Deprivations experienced in urban areas span across institutional deficiencies, poor governance, environmental degradation, and lack of social and economic infrastructures (24–28). With the general poor state of infrastructures and social amenities in most of the SSA urban centres, some typical deprivations experienced by urban women in SSA may include water, sanitation, housing conditions, physical and living environment, and access to health services (29). Available evidence suggests a pathway that multiple dimensions of women's socio-economic disadvantage may have significant influence on family planning utilisation (30).

This study, therefore, aimed to investigate the extent to which women's deprivation status may influence modern family planning utilisation among urban women in West African countries adjusting for potential correlates and confounders. Our approach is guided by existing literature that have established the utility and validity of the index of social and economic deprivations in healthcare utilisation and health outcome research (31, 32).

## Materials and Methods

### Data Source

The study utilised a secondary analysis of the most recent Demographic and Health Survey (DHS) of sexually active women of reproductive age 15–49 years in five West African countries conducted after the inauguration of the Sustainable Development Goals (SDGs). This is a reasonable cut-off time in order to be able to compare results within the same time interval. The analysis was conducted on the pooled sample of 21,641 women of reproductive ages 15–49 years residing in an urban setting in the selected countries: Benin 2017–2018 (*n* = 4,388), Guinea 2018 (*n* = 2,119), Mali 2018 (*n* = 2,013), Nigeria 2018 (*n* = 9,457), and Senegal 2017 (*n* = 3,664). For this study, we adopted DHS classification of urban settings as applicable to each country and defined in the respective country's DHS report. Details of the sampling and data collection techniques are available in the DHS report of each country (33–37).

### Variable Measurements

#### Outcome Variables

Two outcome variables—current use of modern contraceptives (CUMC) and long-acting reversible contraceptives (LARC) use—were examined in this study as binary outcomes, which allowed for assessment of two of the principal dimensions of the family planning programme and utilisation in urban West Africa in the context of relative socioeconomic disadvantages. Though CUMC encompasses all contraceptive methods, this study separately considers LARC use based on the perspective that correct usage and effectiveness of LARC are less dependent on the user's attributes such as educational level, unlike the short-acting reversible methods, which are highly dependent on the attributes (38, 39). Since our study is based on West African women characterised with low levels of education, it is imperative to independently examine the LARC utilisation that accommodates the women's socioeconomic attributes. The first outcome, CUMC, was measured based on the DHS definition as the utilisation of any modern method of family planning such as the pills, IUD, injectables, male condom, female condom, female sterilisation, male sterilisation, implants/Norplant, lactational amenorrhoea, emergency contraception, other modern methods, and standard day method. The second outcome (i.e., LARC) is measured based on whether a respondent who was practising family planning was utilising any of the IUD and implants/Norplant at the time of the survey. Accordingly, those who were using these methods were coded as 1, while those using other methods and non-users were broadly coded as 0. The analyses excluded all sexually active urban women who were not exposed to the risk of pregnancy at the time of the survey including women who were pregnant, infecund, and menopausal.

#### Explanatory Variable

The main explanatory variable is women's deprivation status. A factor score of deprivation index was derived, using principal component analysis, based on 11 indicators: living in poorest/poorer households, having no/primary education, being unemployed; never heard about family planning on radio in the last few months, never heard about family planning on television in the last few months, never read about family planning in newspaper/magazine in the last few months, owns no mobile telephone, never used the Internet, indicating that getting permission for medical treatment is a big problem, getting money for medical treatment is a big problem; and distance to health facility is a big problem. Our approach is guided by existing literature on social and economic deprivations in healthcare utilisation and health outcomes research (31, 32). A reliability analysis test with the Cronbach alpha (α = 0.65) of the indicators indicated an acceptable measure of the deprivation index. Evidence suggests that a reliability test with an alpha value of 0.6–0.7 is acceptable in social and exploratory research (40, 41). The factor scores constituting the extent of deprivation that a respondent is subjected were then classified into three quantile groups in the lower, middle, and upper classes.

Other covariates and confounding factors were also included to obtain the adjusted effect of the respondent's deprivation status on the outcomes. These include respondent's age groups, union status, age at first union, parity, birth intention, religion, and partner's level of education.

### Statistical Analysis

Data were analysed using univariate, bivariate, and multivariable analytical methods. At the univariate level, we used frequency and percentage distributions to examine the prevalence of the outcomes. At the bivariate and multivariable levels, binary logistic regression was used to examine the unadjusted effect of women's deprivation status on the outcomes in model 1, while the adjusted effect was assessed in model 2 by controlling for the potential confounding effects of other selected covariates on the association. The strengths of the associations were assessed using regression coefficients and the standard error. We excluded observations with missing information from the analyses and applied appropriate sample weights as provided in the DHS. Analysis was done using R statistical computing software version 4.0.5 developed and maintained by R Core Team (42).

### Ethical Considerations

This study is based on an analysis of secondary datasets from national household surveys with no respondents' identifiers. The surveys were approved by the countries' National Ethics Committees and implemented by the ICF International in conjunction with relevant agencies in each country. Permission to use the datasets was obtained from the ICF International.

## Results

The background information is presented in Table 1. More than two-fifths of the respondents were in the age groups 25–34 except in Mali (38.2%). Majority (68.2–82.3%) were currently in a union, and more than one-quarter had their first union before their 18th birthday. More than half of the women were multiparous with two or more children, and 7–8 in 10 of them want an additional child later or no more. Muslim women were in the majority in Guinea (88.1%), Mali (98.0%), and Senegal (95.0%), while non-Muslims, mainly the Christians (result not shown), were in the majority in other countries. Except for Nigeria (53.8%), a fewer proportion (24.6–39.4%) of the women's partners had secondary/higher education.

**Table 1:** Sociodemographic distribution of the study samples in the selected West African countries.

	**Benin** ***N =* 4,388**	**Guinea** ***N =* 2,119**	**Mali** ***N =* 2,013**	**Nigeria** ***N =* 9,457**	**Senegal** ***N =* 3,664**	**Pooled data** ***N =* 21,641**
Age group						
15–24	33.5	38.7	37.6	22.8	19.7	26.8
25–34	40.9	40.6	38.2	43.2	42.0	41.9
35–49	25.6	20.7	24.1	34.0	38.3	31.3
Union status/type						
In union	73.6	68.2	78.2	74.8	82.3	75.5
Not in union	26.4	31.8	21.8	25.2	17.7	24.5
Age at first union						
18+/Single	73.0	66.9	61.8	74.5	74.2	72.5
Before 18	27.0	33.1	38.2	25.5	25.8	27.5
Parity						
0–1	34.5	46.6	35.4	32.3	32.6	34.4
2–3	30.2	26.8	29.4	29.6	31.2	29.7
4+	35.2	26.6	35.2	38.0	36.2	35.9
Birth intention						
Later/no more	79.9	69.2	73.2	75.4	75.9	75.6
Sooner	20.1	30.8	26.8	24.6	24.1	24.4
Religion						
Muslim	29.6	88.1	98.0	42.7	95.0	57.7
Non-Muslim	70.4	11.9	2.0	57.3	5.0	42.3
Partner's education						
None	27.9	32.1	34.0	10.2	30.1	20.7
Primary	15.7	4.8	8.5	9.6	17.3	11.5
Secondary/Higher	24.7	29.3	30.2	53.8	24.6	39.4
Not in union/Do not know	31.7	33.9	27.2	26.3	28.0	28.4
**Total**	**4,388 (20.3)**	**2,199 (10.8)**	**2,013 (9.3)**	**9,457 (43.7)**	**3,664 (16.9)**	**21,641**

*Values are in percentages*.

Table 2 presents the socioeconomic deprivation status and the component indicators. Overall, about 3 in 10 of the women were severely deprived with considerable variations among countries. The constituent indicators of the aggregate deprivation measure show that 1 in 10 of the women lives in poor households, about 47.5% attained less than secondary education, while 28.5% were unemployed. More than half of the women had not heard/read about family planning in the few weeks prior to the survey, whereas about one-fifth has no mobile phone, and more than two-thirds had no access to the Internet. Results also indicated that minority of the respondents considered as a big problem getting permission for medical treatment, getting money for treatment, and distance to a health facility.

**Table 2:** Percentage distribution of the respondents by deprivation status and the indicators of the deprivation index in West African countries.

**Socioeconomic deprivation and the indicators**	**Benin**	**Guinea**	**Mali**	**Nigeria**	**Senegal**	**Pooled data**
Deprivation status						
No/Low	24.4	30.6	35.2	40.1	47.3	37.3
Moderate	29.0	38.1	37.0	33.2	35.2	33.5
Severe	46.6	31.2	27.8	26.7	17.4	29.2
Indicators of the deprivation index						
% in poor household	20.3	2.2	0.2	9.6	6.6	9.6
% with < secondary education	65.5	60.7	55.3	29.4	67.6	47.5
% unemployed	22.4	32.9	42.7	24.7	36.6	28.5
% heard no FP information via radio	52.3	61.2	66.6	54.8	38.9	53.0
% read no FP information via mobile phone	69.7	68.5	59.0	65.4	24.9	59.0
% read no FP information via newspaper/magazine	85.8	92.6	92.3	91.7	86.0	89.8
% with no own mobile phone	31.1	9.3	14.4	19.0	13.4	19.0
% with no access to internet	85.0	61.1	60.8	67.4	59.5	68.2
% getting permission for medical treatment a big problem	19.5	17.6	26.3	8.3	3.2	11.6
% getting money for medical treatment a big problem	49.8	46.7	32.4	37.3	38.0	40.2
% distance to health facility a big problem	25.0	23.3	15.6	17.2	8.4	17.6

### Prevalence of Modern Contraceptive and Long-Acting Reversible Contraceptive Use by Deprivation Status of West African Women

The result in Table 3 indicates the modern contraceptive and LARC utilisation prevalence rates among urban women with deprivation status. Overall, less than one-quarter of the urban women were using modern contraceptive (Guinea 30.1%, Mali 31.2%, and Senegal 45.7%). Across the deprivation levels, contraceptive prevalence rates (CPRs) of the modern methods were lowest among the women who were severely deprived: Nigeria (16.0%), Benin (16.5%), Guinea (23.9%), Mali (24.3%), and Senegal (39.8%), and were quite lower than the average urban rate in each country. Women with no/low deprivation had the highest CPR and was higher than the urban averages across the countries.

**Table 3:** Percentage distribution of modern contraceptive use among urban women in West African countries.

	**Benin** ***N =*** **4,388**		**Guinea** ***N =*** **2,119**		**Mali** ***N =*** **2,013**		**Nigeria** ***N =*** **9,457**		**Senegal** ***N =*** **3,664**		**Pooled data** ***N =*** **21,641**	
	**CPR**	**LARC**	**CPR**	**LARC**	**CPR**	**LARC**	**CPR**	**LARC**	**CPR**	**LARC**	**CPR**	**LARC**
**Deprivation status**												
No/Low	29.9	13.7	39.6	16.9	40.7	31.8	29.6	13.2	45.3	30.0	34.7	18.6
Moderate	21.9	14.1	27.4	14.0	27.4	21.8	23.8	13.1	49.0	37.5	28.8	18.5
Severe	16.5	11.5	23.9	9.6	24.3	19.3	16.0	9.1	39.8	32.6	20.0	13.0
Urban average	21.4	12.8	30.1	13.5	31.2	24.6	24.1	12.1	45.6	33.1	28.4	16.9

*CPR, current contraceptive prevalence; LARC, long-acting reversible contraceptive*.

Variations in the prevalence of LARC also follow a similar pattern. Overall, about 12.1–13.5% of the urban women were currently using LARC except in Mali (24.6%) and Senegal (33.1%). By levels of deprivation, women with severe deprivation had the least proportion of users of LARC except in Senegal. However, the disparities between severe deprivation and no/low deprivation groups were substantial in Guinea (9.6 vs. 16.9%) and Mali (19.3 vs. 31.8%), unlike in other countries with negligible differences. The prevalence of LARC among the moderate deprivation group compared with other groups was rather mixed; it was generally higher compared with the severely deprived but lower than the no/low deprivation group in Guinea and Mali.

### Multivariable Analyses

#### Effects of Women Deprivation on Modern Contraceptive Utilisation Among Urban Women in Selected West African Countries

The unadjusted (Model 1) and adjusted (Model 2) regression results of the multivariable analysis in Table 4 present the effect of deprivation on modern contraceptive use, using the pooled and country-specific data. In the pooled data, contraceptive utilisation was significantly and inversely associated with the level of deprivation. Overall, the unadjusted model indicates that the log-odds of using modern contraceptives reduced by 0.27 among the moderately deprived (ß = −0.27, SE = 0.05, *p* < 0.01) and by 0.75 among the severely deprived women (ß = −0.75, SE = 0.06, *p* < 0.01) relative to their counterparts with no/low deprivation (the reference group). This pattern in the unadjusted models of the pooled data is similar across the countries with about 0.30–0.62 decrease in the log-odds of using modern contraceptive among the moderately deprived and about 0.73–0.80 decrease among the severely deprived women compared with the no/low deprivation group, except in Senegal. Adjusting for the covariates (age, union status, age at first union, parity, birth intention, religion, and partner's education) in Model 2, the same patterns of association and statistical significance were sustained in the pooled data and across the countries, except in Senegal where the severely deprived women became associated with 0.43 decrease in the log-odds of using modern contraceptive (ß = −0.43, SE = 0.14, *p* < 0.05), holding other variables in the adjusted model constant.

**Table 4:** Binary logistic regression of the effects of women deprivation on the use of modern contraceptive among urban women in selected West African countries using the pooled data and individual country data.

		**Pooled data**		**Benin**		**Guinea**		**Mali**		**Nigeria**		**Senegal**	
**Characteristics**		**Model 1** **ß(SE)**	**Model 2** **ß(SE)**	**Model 1** **ß(SE)**	**Model 2** **ß(SE)**	**Model 1** **ß(SE)**	**Model 2** **ß(SE)**	**Model 1** **ß(SE)**	**Model 2** **ß(SE)**	**Model 1** **ß(SE)**	**Model 2** **ß(SE)**	**Model 1** **ß(SE)**	**Model 2** **ß(SE)**
Deprivation status	No/Low	0.00	0.00	0.00	0.00	0.00	0.00	0.00	0.00	0.00	0.00	0.00	0.00
	Moderate	−0.27 (0.05)^**^	−0.30 (0.05)^**^	−0.42 (0.11)^**^	−0.46 (0.11)^**^	−0.55 (0.13)^**^	−0.40 (0.13)^*^	−0.60 (0.16)^**^	–−0.62 (0.16)^**^	−0.30 (0.07)^**^	−0.33 (0.07)^**^	0.15 (0.11)	−0.02 (0.10)
	Severe	−0.75 (0.06)^**^	−0.65 (0.06)^**^	−0.77 (0.11)^**^	−0.77 (0.12)^**^	−0.73 (0.18)^**^	−0.61 (0.18)^*^	−0.76 (0.17)^**^	–−0.80 (0.17)^**^	−0.80 (0.10)^**^	−0.68 (0.11)^**^	−0.23 (0.13)	−0.43 (0.14)^*^
**Covariates**													
Age group	25–34		0.00		0.00		0.00		0.00		0.00		0.00
	15–24		−0.13 (0.06)^*^		−0.26 (0.13)^*^		0.001 (0.19)		−0.14 (0.18)		−0.25 (0.10)^*^		−0.15 (0.17)
	35–49		0.01 (0.06)		0.01 (0.11)		0.23 (0.16)		−0.20 (0.17)		0.09 (0.09)		−0.15 (0.12)
Union status/type	In union		0.00		0.00		0.00		0.00		0.00		0.00
	Not in union		−0.17 (0.12)		0.42 (0.26)		0.67 (0.36)		−0.15 (0.33)		0.50 (0.42)		−1.07 (0.22)^**^
Age at first union	18+/Single		0.00		0.00		0.00		0.00		0.00		0.00
	Before 18		−0.09 (0.05)		0.16 (0.11)		0.03 (0.12)		−0.18 (0.14)		−0.000 (0.09)		−0.26 (0.12)^*^
Parity	0–1		0.00		0.00		0.00		0.00		0.00		0.00
	2–3		0.24 (0.06)^**^		0.22 (0.12)		−0.11 (0.20)		0.26 (0.21)		0.17 (0.09)		0.51 (0.13)^**^
	4+		0.43 (0.08)^**^		0.51 (0.15)^**^		0.06 (0.24)		0.54 (0.26)^*^		0.28 (0.11)^*^		0.76 (0.19)^**^
Birth intention	Later/No more		0.00		0.00		0.00		0.00		0.00		0.00
	Sooner		−1.00 (0.06)^**^		−0.54 (0.13)^**^		−0.32 (0.16)^*^		−1.02 (0.18)^**^		−1.09 (0.10)^**^		−1.47 (0.12)^**^
Religion	Muslim		0.00		0.00		0.00		0.00		0.00		0.00
	Non-Muslim		0.15 (0.05)^*^		0.04 (0.11)		−0.04 (0.21)		−0.04 (0.31)		0.20 (0.07)^*^		0.43 (0.27)
Partner's education	None		0.00		0.00		0.00		0.00		0.00		0.00
	Primary		0.34 (0.08)^**^		0.19 (0.16)		−0.46 (0.30)		−0.11 (0.19)		0.35 (0.18)		0.74 (0.16)^**^
	Secondary/Higher		0.42 (0.07)^**^		0.39 (0.15)^*^		0.20 (0.16)		0.42 (0.18)^*^		0.62 (0.17)^**^		0.29 (0.16)
	Not in union/Do not know		0.25 (0.11)^*^		0.26 (0.24)		0.24 (0.35)		0.38 (0.28)		−0.46 (0.41)		0.26 (0.18)
Country	Benin		0.00		na		na		na		na		na
	Guinea		0.69 (0.12)^**^		na		na		na		na		na
	Mali		0.64 (0.11)^**^		na		na		na		na		na
	Nigeria		−0.01 (0.07)		na		na		na		na		na
	Senegal		1.13 (0.09)^**^		na		na		na		na		na
**Model diagnostics**													
	Observations	21,641		4,388		2,119		2,013		9,457		3,664	
	Log Likelihood	−12,862.00	−12,047.00	−2,231.20	−2,184.40	−1,353.60	−1,291.70	−1,188.80	−1,135.10	−5,257.60	−4,995.00	−2,408.80	−2,130.20
	Akaike Inf. Crit.	25,730.00	24,130.00	4,468.40	4,396.70	2,713.10	2,611.40	2,383.60	2,298.20	10,521.00	10,018.00	4,823.60	4,288.40

* *p < 0.05*;

** *p < 0.001; na, not applicable*.

Examining the roles of the covariates in the adjusted model of the pooled data, the log-odds of modern contraceptive use decreases by 0.13 for women who are of the age group 15–24 (ß = −0.13, SE = 0.06 *p* < 0.05), by 1.00 for women who want another child sooner (ß = −1.00, SE = 0.06, *p* < 0.01), but increases by 0.24–0.43 for multiparous women (parity 2–3: ß = 0.24, SE = 0.06, *p* < 0.01; and parity 4+: ß = 0.43, SE = 0.08, p < 0.01), by 0.15 for the non-Muslims (ß = 0.15, SE = 0.05, *p* < 0.05), and by 0.34–0.42 for the women whose husbands were educated (primary: ß = 0.34, *p* < 0.01; and secondary/higher: ß = 0.42, *p* < 0.01) relative to their counterparts in the respective reference groups. However, the results across countries are mixed. While the predicted log-odds of contraceptive use among women who want another child sooner compared with those who want later/no more consistently decrease across the countries, that of other covariates were significant only in a few countries.

#### Effects of Women Deprivation on Long-Acting Reversible Contraceptive Utilisation Among Urban Women in Selected West African Countries

The results of the multivariable analysis of the association between women deprivation and LARC utilisation in Table 5 follow similar patterns with that of the modern contraceptive use in Table 4, with a few exceptions. As indicated, severe deprivation had negative effect on LARC use among the respondents. In the unadjusted model (Model 1) of the pooled data, severely deprived women had 0.42 decrease in the log-odds of using LARC (ß = −0.42, SE = 0.07, *p* < 0.05) compared with the no/low deprivation group. Except in Benin and Senegal, this pattern in the unadjusted model is consistent in other countries with 0.42–0.67 decrease in the log-odds of LARC use by the severely deprived women compared with those with no/low deprivation. Adjusting for the covariates in Model 2, the severely deprived women sustained the reduced log-odds of using LARC in the pooled data (ß = −0.44, SE = 0.07, *p* < 0.01), and across the countries with 0.42–0.72 decrease, except Senegal.

**Table 5:** Binary logistic regression of the effects of women deprivation on use of long-acting reversible contraceptives (LARC) among urban women in selected West African countries, using the pooled data and individual country data.

		**Pooled data**		**Benin**		**Guinea**		**Mali**		**Nigeria**		**Senegal**	
**Characteristics**		**Model 1** **ß(SE)**	**Model 2** **ß(SE)**	**Model 1** **ß(SE)**	**Model 2** **ß(SE)**	**Model 1** **ß(SE)**	**Model 2** **ß(SE)**	**Model 1** **ß(SE)**	**Model 2** **ß(SE)**	**Model 1** **ß(SE)**	**Model 2** **ß(SE)**	**Model 1** **ß(SE)**	**Model 2** **ß(SE)**
Deprivation status	No/Low	0.00	0.00	0.00	0.00	0.00	0.00	0.00	0.00	0.00	0.00	0.00	0.00
	Moderate	−0.003 (0.06)	−0.10 (0.06)	0.03 (0.14)	−0.15 (0.14)	−0.22 (0.17)	−0.07 (0.18)	−0.52 (0.15)^*^	−0.52 (0.16)^*^	−0.01 (0.11)	−0.18 (0.11)	0.34 (0.12)^*^	0.15 (0.12)
	Severe	−0.42 (0.07)^**^	−0.44 (0.07)^**^	−0.21 (0.14)	−0.42 (0.15)^*^	−0.65 (0.21)^*^	−0.53 (0.22)^*^	−0.67 (0.18)^**^	−0.72 (0.18)^**^	−0.42 (0.12)^**^	−0.61 (0.13)^**^	0.12 (0.13)	−0.14 (0.15)
**Covariates**													
Age group	25–34		0.00		0.00		0.00		0.00		0.00		0.00
	15–24		−0.11 (0.07)		−0.53 (0.18)^*^		0.02 (0.20)		−0.11 (0.17)		−0.15 (0.15)		−0.21 (0.17)
	35–49		−0.04 (0.05)		0.08 (0.11)		0.47 (0.20)^*^		−0.25 (0.19)		0.05 (0.09)		−0.33 (0.12)^*^
Union status/type	In union		0.00		0.00		0.00		0.00		0.00		0.00
	Not in union		−0.52 (0.13)^**^		−0.02 (0.28)		0.42 (0.46)		0.10 (0.37)		−0.39 (0.51)		−1.25 (0.20)^**^
Age at first union	18+/Single		0.00		0.00		0.00		0.00		0.00		0.00
	Before 18		−0.02 (0.06)		0.30 (0.12)^*^		−0.07 (0.18)		−0.07 (0.16)		0.15 (0.11)		−0.17 (0.12)
Parity	0–1		0.00		0.00		0.00		0.00		0.00		0.00
	2–3		0.58 (0.08)^**^		0.61 (0.17)^**^		−0.14 (0.23)		0.19 (0.23)		1.21 (0.19)^**^		0.58 (0.16)^**^
	4+		0.86 (0.09)^**^		0.78 (0.21)^**^		0.03 (0.27)		0.43 (0.29)		1.47 (0.20)^**^		0.92 (0.18)^**^
Birth intention	Later/No more		0.00		0.00		0.00		0.00		0.00		0.00
	Sooner		−1.03 (0.07)^**^		−0.57 (0.15)^**^		−0.45 (0.19)^*^		−0.97 (0.18)^**^		−1.06 (0.13)^**^		−1.31 (0.13)^**^
Religion	Muslim		0.00		0.00		0.00		0.00		0.00		0.00
	Non-Muslim		−0.06 (0.08)		−0.22 (0.14)		0.15 (0.21)		−0.68 (0.45)		0.08 (0.10)		−0.002 (0.36)
Partner's education	None		0.00		0.00		0.00		0.00		0.00		0.00
	Primary		0.26 (0.08)^*^		0.15 (0.18)		−0.30 (0.38)		−0.53 (0.25)^*^		0.23 (0.21)		0.37 (0.14)^*^
	Secondary/Higher		0.33 (0.07)^**^		0.25 (0.17)		0.36 (0.20)		0.23 (0.17)		0.36 (0.17)^*^		0.16 (0.14)
	Not in union/Do not know		0.17 (0.13)		0.12 (0.25)		0.36 (0.47)		0.27 (0.30)		−0.52 (0.49)		0.14 (0.19)
Country	Benin		0.00		na		na		na		na		na
	Guinea		0.22 (0.13)		na		na		na		na		na
	Mali		0.80 (0.13)^**^		na		na		na		na		na
	Nigeria		−0.23 (0.09)^*^		na		na		na		na		na
	Senegal		1.12 (0.11)^**^		na		na		na		na		na
**Model diagnostics**													
	Observations	21,641		4,388		2,119		2,013		9,457		3,664	
	Log Likelihood	−9,872.70	−8,888.00	−1,682.20	−1,598.10	−895.42	−863.12	−1,078.10	−1,034.40	−3,564.00	−3,174.40	−2,219.10	−1,976.00
	Akaike Inf. Crit.	19,751.00	17,812.00	3,370.40	3,224.20	1,796.80	1,754.20	2,162.10	2,096.70	7,134.10	6,376.90	4,444.20	3,980.10

* *p < 0.05*;

** *p < 0.001; na, not applicable*.

The table also indicates a few covariates, which play important roles in the adjusted model of the pooled data, though in different ways. While union status and birth intention are inversely related to LARC use, parity and partner's education were positively related. On one hand, in the pooled data, the log-odds of using LARC decreases by 0.52 among the women who were not in union (ß = −0.52, SE = 0.13, *p* < 0.01) and by 1.03 for women who wanted another child sooner (ß = −1.03, SE = 0.07, *p* < 0.01) compared with their respective reference groups. This pattern of LARC use is significant for women who are not in union only in Senegal (ß = −1.25, SE = 0.20, *p* < 0.01), whereas, women who wanted another child sooner had similar odds across the countries. On the other hand, multiparous women had about 0.58–1.47 decrease in the log-odds of using LARC in the pooled data (parity 2–3: ß = 0.58, SE = 0.08, *p* < 0.01; parity 4+: ß = 0.86, SE = 0.09, *p* < 0.01) and across the countries except Guinea and Mali. Women with educated partner had similar odds in the pooled data and in a few countries: Nigeria and Senegal.

## Discussion

This study reiterates the importance of social inequalities in health care utilisation and outcomes among urban women in West Africa. It highlights the key findings with respect to women deprivation in the urban geographies in relation to family planning utilisation among urban women in the selected West African countries. This underscored the deprivation context of urban geographies, particularly among women within the urban space, in family planning intervention programmes and policies.

Our findings showed that substantial urban women in the West African sub-region are still largely socially and economically disadvantaged, even though they are erroneously assumed to be better than those in rural geographies (22, 43). Although, there is convergence of evidence that urban geographies fair better in terms of health care utilisation and outcomes compared with rural areas (21), our findings show that such dichotomy beclouds some of the realities particularly among women in urban locations. Women deprivations exerts some limitation on women's ability to utilise health services including family planning. Rapid urbanisation in the West African sub-region, with associated pressures on the socioeconomic and ecological resources, may have dire consequences on the worsening urban inequalities (44).

Our findings showed that the utilisation of modern family planning is still low in the urban centres of the sub-region. Non-utilisation, as observed in the existing studies, may be linked to several reasons including some myths and ideational factors (45–47), which may be further heightened by deprivation and lack of access to correct information. Although, many scholars in the sub-region have advocated for policies targeted toward improved family planning uptake in the rural areas (48–50), our findings suggest the need for more intense attention on urban centres where the relatively better social and health care services are accessed disproportionately.

We found that the social and economic inequalities among urban women play important roles in family planning utilisation. Not only was a strong association observed between the women's deprivation levels and modern contraceptive use, but also the hypothesised dose-response relationship between the extent of deprivation and contraceptive use was remarkably discernible in both the pooled samples and for each country. Our findings further confirmed previous findings that deprived women tend to have low contraceptive uptake (31) and adverse health outcomes (32). Women who are deprived in basic social and economic indicators—household wealth status, employment, access to family planning information, autonomy, and access to a healthcare facility—have often been equally deprived of access to quality and adequate reproductive health care services (51, 52).

Though our study did not consider the individual effects of the deprivation components, the results strongly echo their negative influence on contraceptive uptake as having been extensively documented in previous studies (53–57). For instance, studies have shown significant disparities in the use of modern contraceptives favouring women from rich households over those from poor households (53, 54, 57). Moreover, low contraceptive use have also been found among women who reported financial and geographic impediments to family planning services (58, 59). More importantly, education and family planning message exposure increases women agency, facilitate diffusion of information, expands women's choices, promote behavioural changes, and improve participation in the use of modern contraceptives (53, 54, 57, 60, 61). Education and targeted information dispel myths, misinformation, and fears held against contraceptives.

Our findings further indicate that the severely deprived women were less likely to utilise LARC, though the contraceptive methods are more effective than the short-acting ones. This may imply failed contraceptive experiences, resulting in unintended pregnancy. LARCs are more expensive than the short-acting methods and may be unaffordable for the poor, thus, the low utilisation among the severely deprived urban women. Poor LARC utilisation in our study also suggests some interplay between contraceptive demand and supply. Demand for and supply of contraceptive methods are often positively related (62). Hence, more expensive contraceptive methods may have lower demand and, consequently, low supply, which worsens low LARC uptake in a disadvantaged setting.

We also observed country disparities in the effect of women's deprivation status on LARC utilisation. Remarkably, while moderate or severe deprivation among the women showed a significant adjusted effect on LARC utilisation in other West African countries, no significant effect was observed in Senegal (Table 5). This disparity may be linked to the higher prevalence of modern contraceptive utilisation among Senegalese women compared with other West African countries included in this study. We attribute the lack of consistencies in the region's prevalence of modern contraceptive use to possible effects of age at marriage.

We found that, apart from women deprivation, some demographics such as age, union status, birth intention, and parity also influence family planning uptake among urban women. Some of the demographics importantly play the role of sustaining the deprivation status of the women. For instance, women aged 15–24 were found to have low contraceptive uptake, which may have significant implication in the region with a booming youth population.

Interestingly, we found that women who were not in union were less likely to use modern contraceptive in Senegal. This finding is divergent from the existing studies, which associated higher contraceptive use to unmarried women compared with the married (63). The low contraceptive uptake among the Senegalese women who were not in union is expected, based on the lower proportion of the deprived women in the country. In addition, the estimate may be linked to the reduced sexual activities among the unmarried Senegalese whose premarital sexual activities are being censored by the country's cultural and religious norms (64). Also, the proportions of urban women that are utilising modern contraceptives are higher among those whose partners have higher education. This further ascertain men's role in women achieving desired fertility (65, 66).

Our findings support existing evidence on the myriads of sexual and reproductive health challenges confronting disadvantaged women populations in urban settings in sub-Saharan Africa (5, 67, 68). Coupled with their high concentration in the informal urban settlements, the highly deprived urban women often had to contend with unequal access to and high unmet needs for modern family planning services, especially the long-acting reversible methods that offer better protection against unwanted pregnancies and unsafe abortion (5, 69).

For instance, a recent study by Tetui et al. (68) in Kira Municipality of Uganda contextualises the gross spatial inequalities in access, distribution, and quality of modern family planning services between formal and informal settings with a greater disparity in access to long-acting reversible methods. Nonetheless, the dominance of the private sector in family planning service provision in informal settlements and the attendant higher out-of-pocket service fees might impose serious constraints not only on the uptake but also on the sustained use of modern family planning among the highly deprived urban women (5). Such supply-side constraints usually coexist with other knowledge, attitude, and sociocultural barriers operating on the demand side.

### Study Limitations

This study is not without limitations. Being a cross-sectional study, causation could not be established between the explanatory variables, the women deprivation, as well as the covariates and the outcome variables. Also, since the study was based on secondary data sources, no additional variables could be added beyond what the data contained. For instance, other relevant covariates that could be included in the model but were not available in the datasets include reasons for not using LARC methods. Besides, the study was based on only five West African countries with post-SDG standard demographic and health survey data addressing the outcome variables of interest. These limitations, however, do not in any way compromise the validity and reliability of the study; they only suggest the need for caution in the interpretation of the findings.

There are noticeable programs and policy changes in some of these countries that may influence some positive changes in the nearest future. Many countries in the region are making more commitments to support family planning with a dedicated budget since the London Family Planning Summit 2017 (70). Also, there are increasing funder support and new innovative initiatives toward improving family planning in urban geographies particularly with the Challenge Initiatives and a few other programs (71). There is the prospect of improvement in family planning utilisation and access with sustained government commitment and partner's support.

## Conclusion

Inequalities among women in urban areas constitute a major factor in health care utilisation. The combined effect of women deprivation and other sociodemographic gradients have significant effect on family planning utilisation among urban women, particularly those living in informal settlements. Programmes and policy efforts need to factor in these disparities.

## Data Availability Statement

The dataset presented in this study can be found in the online repository of the DHS program: https://dhsprogram.com/data/available-datasets.cfm.

## Author Contributions

AA, JM, and OI formulated the background for the study. JM and EI formulated the Materials and Methods for the study, as well as the Results, and with JA wrote the Discussion. AA, JM, and EI reviewed the manuscript. EI performed data analysis. JA and OI reviewed the literature and OI created the references. All authors contributed to the article and approved the submitted version.

## Conflict of Interest

The authors declare that the research was conducted in the absence of any commercial or financial relationships that could be construed as a potential conflict of interest.
